# AI-Based Prediction of Ultrasonic Vibration-Assisted Milling Performance

**DOI:** 10.3390/s24175509

**Published:** 2024-08-26

**Authors:** Mohamed S. El-Asfoury, Mohamed Baraya, Eman El Shrief, Khaled Abdelgawad, Mahmoud Sultan, Ahmed Abass

**Affiliations:** 1 Department of Production Engineering and Mechanical Design, Faculty of Engineering, Port Said University, Port Fuad 42526, Egypt; mohamed.saad@eng.psu.edu.eg (M.S.E.-A.); mohamed.baraya@eng.psu.edu.eg (M.B.); eman.ahmed@eng.psu.edu.eg (E.E.S.); k.abdelgawad95@eng.psu.edu.eg (K.A.); mahmoud.sultan@eng.psu.edu.eg (M.S.); 2 Department of Materials, Design and Manufacturing Engineering, School of Engineering, University of Liverpool, Liverpool L69 3GH, UK

**Keywords:** artificial intelligence, support vector regression, neural network, ultrasonic, vibration assisted cutting, milling, titanium, aluminium

## Abstract

The current study aims to evaluate the performance of the ultrasonic vibration-assisted milling (USVAM) process when machining two different materials with high deviations in mechanical properties, specifically 7075 aluminium alloy and Ti-6Al-4V titanium alloy. Additionally, this study seeks to develop an AI-based model to predict the process performance based on experimental data for the different workpiece characteristics. In this regard, an ultrasonic vibratory setup was designed to provide vibration oscillations at 28 kHz frequency and 8 µm amplitude in the cutting feed direction for the two characterised materials of 7075 aluminium alloy (150 BHN) and Ti-6Al-4V titanium alloy (350 BHN) workpieces. A series of slotting experiments were conducted using both conventional milling (CM) and USVAM techniques. The axial cutting force and machined slot surface roughness were evaluated for each method. Subsequently, Support Vector Regression (SVR) and artificial neural network (ANN) models were built, tested and compared. AI-based models were developed to analyse the experimental results and predict the process performance for both workpieces. The experiments demonstrated a significant reduction in cutting force by up to 30% and an improvement in surface roughness by approximately four times when using USVAM compared to CM for both materials. Validated by the experimental findings, the ANN model accurately and better predicted the performance metrics with RMSE = 0.11 µm and 0.12 N for Al surface roughness and cutting force. Regarding Ti, surface roughness and cutting force were predicted with RMSE of 0.12 µm and 0.14 N, respectively. The results indicate that USVAM significantly enhances milling performance in terms of a reduced cutting force and improved surface roughness for both 7075 aluminium alloy and Ti-6Al-4V titanium alloy. The ANN model proved to be an effective tool for predicting the outcomes of the USVAM process, offering valuable insights for optimising milling operations across different materials.

## 1. Introduction

There is an increasing demand for precise, miniaturised products with tight tolerances and high-quality surface finishes across various industries. Titanium alloys, particularly Ti-6Al-4V, are extensively used in aerospace, automotive and medical sectors due to their exceptional strength, lightweight nature, corrosion resistance and biocompatibility [1,2,3]. However, their high strength, low thermal conductivity and reactive chemical properties can lead to significant tool wear during machining under thermal stress. In contrast, aluminium alloys such as Al7075 are widely employed in aerospace, automotive, machinery and medical fields. While Al7075 is valued for its superior strength and corrosion resistance [4,5], it presents machinability challenges, including surface roughness and geometrical accuracy compared to other aluminium alloys, complicating the manufacturing process [6,7]. Ultrasonic vibration-assisted milling (USVAM) is an advanced machining technique that combines conventional milling (CM) with ultrasonic vibrations, significantly improving surface quality [8,9]. The ultrasonic-assisted milling (USAM) process builds on this by enhancing machining performance in several critical areas. It improves surface accuracy, reduces surface roughness, minimises wear and thermal stress and extends tool life. These advancements lead to better quality finishes and reduced operational costs, making USVAM a valuable technique in advanced manufacturing [10,11]. As a result, there has been increased interest in developing and optimising VAM techniques to further enhance machining efficiency and process parameters.

Hu et al. [12] carried out experiments comparing ultrasonic-assisted milling (UAM) to CM of titanium alloy. Their findings showed that UAM greatly reduced chip adhesion during cutting, which led to less burr formation and improved surface integrity. Additionally, UAM achieved a surface roughness of 96 nm, a 46.7% improvement over the 180 nm roughness observed with CM [12]. Liu et al. [13] compared CM and UVM regarding their effects on chip formation and machining quality. Their tests demonstrated that the UVM improves shear slip and chip evacuation, resulting in a smaller chip curl angle. Engelking et al. [14] optimised surface residual stress in additively manufactured Ni alloy components using UAM. Their results indicated that CM primarily induced tensile residual stress, while UAM generated compressive residual stress on the machined surface. Furthermore, their study showed that UAM significantly improved Ni alloy components’ processability and surface integrity, especially at lower cutting speeds. Fang et al. [15] investigated the impact of conventional micro-milling and one-dimensional ultrasonic-assisted vibration micro-milling (UVAMM) on the surface quality of workpieces. Their findings indicated that UVAMM reduced the cutting forces in the three principal directions, F_x_, F_y_ and F_z_, by 7.6%, 11.5% and 1.3%, respectively. Additionally, when the per tooth feed rate (f_z_) was set to 5 µm, the burr-inhibition effect was most pronounced, with the minimum burr sizes measuring 50.23 µm on the down-milling side and 36.57 µm on the up-milling side. Zhang et al. [10] investigated the impacts of CM and UAM on titanium alloy’s surface and subsurface characteristics. They discovered that UAM produced a consistent micro-texture on the surface but resulted in higher surface roughness compared to CM. Additionally, UAM was found to intensify changes in the subsurface structure and cause greater plastic deformation. Xu et al. [16] applied longitudinal-torsional ultrasonic vibration milling (LTUVM) to address subsurface damage and severe burr formation in the production of CFRP materials. They developed the first numerical model for cutting aramid fibre-reinforced polymer (AFRP). Their tests demonstrated that LTUVM reduced burr length by 23–38%. The most significant improvement in burr length, up to 38%, occurred at a cutting speed of 4000 rpm. Additionally, at a speed of 2800 rpm, the cutting force reduction rate was approximately 48%.

Wang et al. [17] analysed the impact of ultrasound-assisted dry spiral milling processing parameters on CFRP delamination damage. Their experimental results revealed that the influence of ultrasound amplitude was relatively minor, accounting for only 18.1% of the overall effect. Amini and Bayat [18] investigated the impact of axial ultrasonic-assisted milling (UAM) on the distortion of aluminium alloy parts. Their study compared the effects of CM and UAM on distortion and milling force. The experiments demonstrated that UAM significantly reduces milling force and distortion compared to CM, showing a 29% reduction in cutting force and a 21% reduction in distortion. This improvement has crucial implications for enhancing the flatness tolerance of workpieces, resulting in components free from waves and warpages. El-Hofy and Ali [19] studied the effect of vibration-assisted helical milling (VAHM) on machining 7075 aluminium alloy. Their experiments showed that applying axial ultrasonic vibrations improved the surface finishing performance. The optimal conditions were a rotational speed of 5000 rpm, a tangential feed of 100 mm/min and a helical pitch of 0.1 mm/rev. Under these conditions, VAHM reduced axial cutting forces and diameter deviation, enhancing overall milling performance. Haddad et al. [20] studied USVAM on AISI 1045 steel and 7075 aluminium, using resonant frequencies of 19,840 and 19,757 Hz, respectively, and increasing ultrasonic intensity from 15 to 22.5 W/cm^2^ improved surface roughness (Rz) by 70% and smoothness (Ra) by 72% for aluminium. For steel, 22.5 W/cm^2^ vibrations enhanced surface smoothness by 53% (average) and 45% (maximum), while 30 W/cm^2^ vibrations increased smoothness by 35% and 25% on face-milled surfaces. Baraya et al. [21] developed a vibratory system for slotting using ultrasonic vibrations at 34.7 kHz and 10 μm amplitude on Al 7075 alloy. Their experiments showed up to a 50% reduction in cutting force and improved surface roughness with ultrasonic vibrations, which also reduced slot width error compared to conventional milling. Their developed statistical model accurately predicted process performance, confirming the technique’s effectiveness for high-precision manufacturing.

The implementation of artificial intelligence (AI) based machine learning (ML) algorithms in milling processes has significantly advanced the monitoring and prediction of factors like tool wear, failure and process optimisation—areas where conventional methods often fall short [22]. Commonly used ML algorithms include Support Vector Machine (SVM) [23], artificial neural networks (ANN) [24] and Decision Trees, with specific subtypes like Probabilistic Neural Networks (PNN) [25] and Random Forest (RF) also being prevalent [26]. These algorithms have been particularly effective in tool condition monitoring, process parameter optimisation and enhancing product quality by predicting surface roughness, cutting force and workpiece deformation [22]. In the field of ultrasonic-assisted milling, which involves costly tools and time-consuming experiments, AI methods, particularly ANNs, offer a practical alternative for predicting the effects of various parameters on cutting processes. ANNs have a strong track record in manufacturing for tasks like process optimisation and tool condition monitoring, making them a reliable choice for modelling ultrasonic-assisted milling (UAM) processes [27]. The ANN model has demonstrated high accuracy in predicting surface roughness for CNC face milling components, and its application in the design of experiments for various machining operations further validates its effectiveness [28]. Concerning the use of AI and machine learning for the prediction and evaluation of milling process performance, Zerti et al. [29] analysed the effectiveness of response surface methodology (RSM) versus artificial neural networks (ANN) in predicting outcomes. Their findings revealed that the ANN model achieved a prediction accuracy of 99.9% for surface roughness and cutting forces.

In contrast, the RSM model provided an accuracy of 87.31% for surface roughness and 98.03% for cutting force values. Karabulut [30] investigated the optimisation of cutting forces and surface roughness in AA7039/Al_2_O_3_ metal matrix composites milling using an ANN model. The study’s results indicated that the ANN model was highly effective, achieving prediction accuracies of 97.75% for surface roughness and 93.34% for cutting forces. Pourmostaghimi et al. [31] devised an advanced methodology utilising ANN to estimate machining parameters in the turning process of AISI D2. Their findings demonstrated that this approach could predict machining time with high accuracy.

This study explores the performance analysis and predictive capabilities of artificial neural networks (ANN) in ultrasonic-assisted milling (UAM) across various material properties. This research addresses the limitations of traditional milling processes by aiming to improve machining precision. An innovative USVAM system was designed to introduce ultrasonic vibrations to the workpiece along the feed direction. Subsequently, a statistical model was developed to predict the responses of the USVAM process, which was validated through additional experimental trials. The resulting ANN model is a tool for effectively controlling the USVAM process, considering the applied vibrations and machining parameters. This advancement demonstrates significant applicability in manufacturing industries that demand precision and high accuracy.

## 2. Methods

### 2.1. FEA Model Construction and Ultrasonic Vibratory Transducer Design

Finite Element Analysis (FEA) is pivotal in accurately simulating the behaviour of complex manufacturing systems [32,33]. Its ability to model intricate geometries and material properties is advantageous for analysing ultrasonic vibratory transducers. FEA allows for detailed simulations of complex vibratory behaviours, enabling the optimisation of transducer performance before building the physical parts without extensive physical prototyping. This approach helps identify potential issues and refine designs early in development, reducing costs and time. Additionally, FEA can accurately predict the effects of different materials and geometries, ensuring the transducer meets specific performance criteria [34]. The FEA’s role in this study was to verify and refine the proposed design. Following this, the components of the finalised design were manufactured. Additionally, FEA was employed to assess the nodal plan within the workpiece. This position was crucial for workpiece fixation during milling, as the wrong fixing position degenerates the vibration waves.

**Figure 1:**
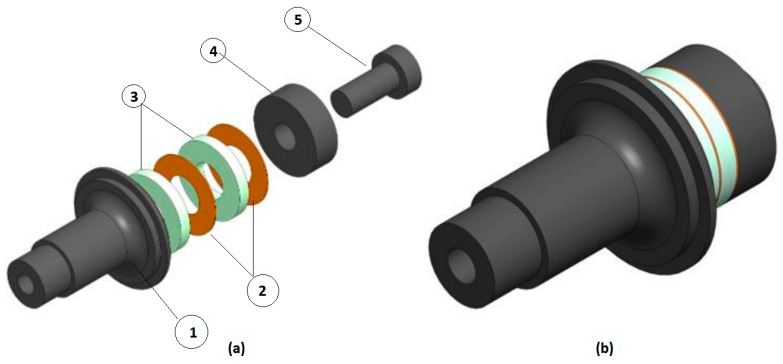
(**a**) Transducer components: (1) front mass, (2) piezoelectric rings, (3) electrodes, (4) back mass, (5) steel bolt and (**b**) Assembled transducer.

The initial step in developing this study’s FEA model involved creating the transducer components’ geometry. The dimensions for each component were determined according to the methodology outlined by Baraya et al. [35]. The ABAQUS 13.1 software package (Dassault Systèmes, Vélizy-Villacoublay, France) was employed to construct the model geometry, as illustrated in Figure 1. Notably, the innovative approach of using FEA allows for precise analysis of the piezoelectric transducer components (front mass, piezoelectric rings, electrodes, back mass and steel bolt), depicted in Figure 1a, which are assembled and held together through a compressive pre-load using a steel bolt.

**Figure 2:**
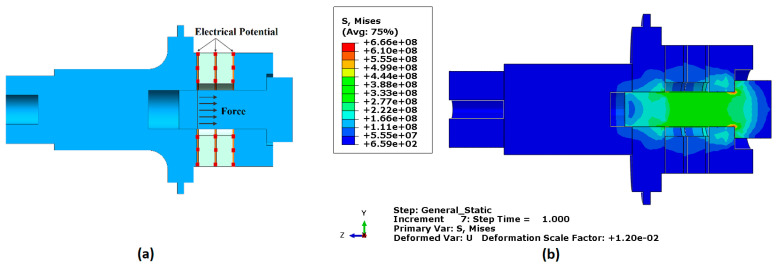
(**a**) Load and boundary conditions and (**b**) von Mises equivalent stress results from finite element static analysis for the transducer.

Following the literature [35], appropriate materials were designated for each of the transducer parts. Aluminium was selected for both the front and back masses due to its lightweight and strength properties. Copper was chosen for the electrodes because of its excellent electrical conductivity. Piezoelectric materials were used for the rings to efficiently transform electrical energy into mechanical energy. Table 1 and Table 2 were used to define the characteristics of these materials in the FEA model. Subsequently, loads and boundary conditions were applied. A pre-stressed bolt load, calculated based on the recommended compression state of the piezoelectric stack (approximately 30 MPa), was applied between the back mass and the piezoelectric stack at the bolt cross-section [35]. Additionally, the boundary conditions (BC) are applied to the piezoelectric elements, where the electrical potential starts from the initial state at the surfaces between the piezoelectric rings themselves and between the piezoelectric rings and the neighbouring metal masses [36,37]. A depiction of these load and boundary conditions can be observed in Figure 2a.

The FEA mesh design for ultrasonic transducers involved careful selection of mesh elements to precisely capture each component’s physical behaviour. All metallic parts were meshed using 3D stress quadratic elements (C3D20R) to ensure detailed stress distribution and deformation characteristics. Piezoelectric quadratic elements (C3D20RE) were chosen for the piezoelectric rings. This selection allows for accurately simulating the electromechanical coupling effects inherent in piezoelectric materials.

### 2.2. FEA Model Outcome

The FEA results, depicted in Figure 2b, verified that the applied bolt torque does not induce high-stress levels that could fail transducer components. This verification ensures the structural integrity and reliability of the transducer under operational conditions. Therefore, the design is robust against the applied torque, safeguarding the transducer’s performance.

The choice of C3D20R elements in Abaqus for metallic parts was successful, as 20-node quadratic brick elements accurately capture curved geometries and stress gradients. Their reduced integration helps avoid common numerical issues, making them more efficient and less computationally intensive. C3D20R elements also perform well in nonlinear analyses, efficiently handling large deformations and complex material behaviours. Their higher-order interpolation improves convergence, meaning fewer iterations for the solver and quicker results. The piezoelectric ring C3D20RE elements enhanced hourglass control and improved numerical stability and accuracy. They excel in nonlinear analyses, capturing deformations and dynamic behaviours essential for optimising transducer performance.

**Figure 3:**
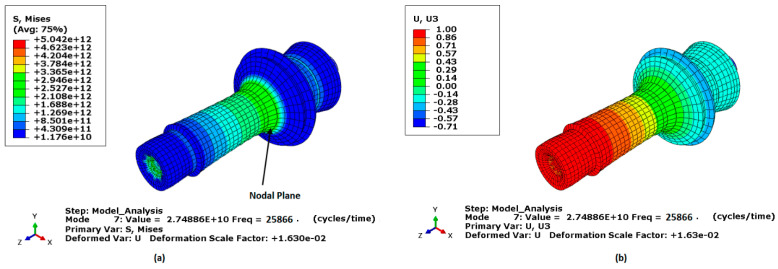
Longitudinal mode shape of the transducer, (**a**) normalised displacement, (**b**) and von Mises stress.

Given that the design frequency was 28 kHz, the frequency analysis was conducted over a 26–30 kHz frequency range. Figure 3 depicts the transducer operating in the longitudinal vibration mode, wherein each particle’s displacement occurs in the axial direction. The measured resonance frequency, at which the transducer naturally oscillates, was 25,866 Hz, which deviates by approximately 7.62% from the design frequency, thus confirming the accuracy of the design. Figure 3a,b present the von Mises stress and displacement distributions at longitudinal mode shape, respectively. These figures illustrate the variations in displacement and stress, highlighting regions of maximum intensity. Notably, the maximum stress was observed at the nodal point (Figure 3a), which was critical for the transducer’s performance during experiments. In contrast, the maximum displacement was recorded at the front-end face of the transducer, Figure 3a,b.

**Figure 4:**
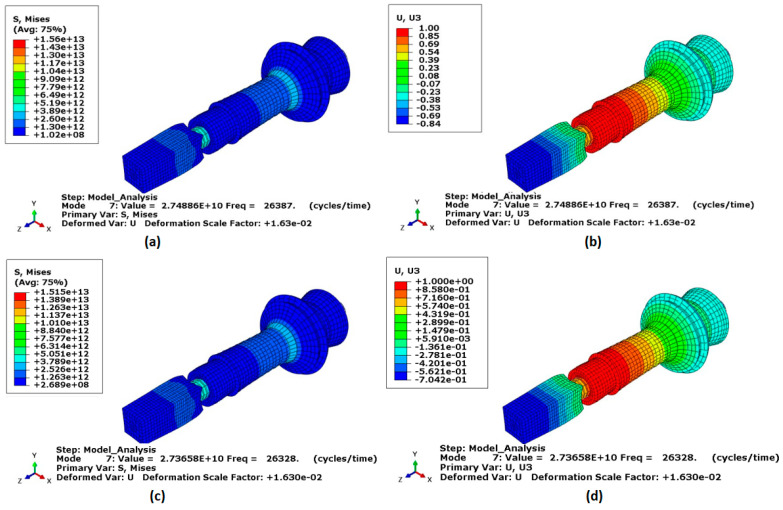
Longitudinal mode shape of the workpiece under the influence of an ultrasonic vibration-assisted milling device. (**a**,**b**) von Mises stress and normalised displacement, respectively, for the Al 7075 alloy workpiece. (**c**,**d**) von Mises stress and normalised displacement, respectively, for the Ti-6Al-4V titanium workpiece.

Figure 4a,b displays the von Mises stress and displacement distributions along the transducer’s axial direction for the longitudinal mode shape after incorporating the horn and workpiece. The resonance frequency for this configuration was measured at 26.387 kHz, which deviates by approximately 5.76% from the design frequency for the Al7075 workpiece, thus further validating the accuracy of the design. Additionally, the resonance frequency for the Ti-6Al-4V titanium workpiece was measured at 26.328 kHz, which deviates by approximately 6% from the design frequency. The frequency error ratio of a transducer refers to the difference between the designed resonant frequency and the actual resonant frequency during operation. This ratio ensures the transducer performs as intended, especially in applications requiring precise ultrasonic frequencies. The error was due to practical constraints and operational tolerances in this case. Cost considerations and empirical data supporting acceptable performance at this error level validate its use.

**Figure 5:**
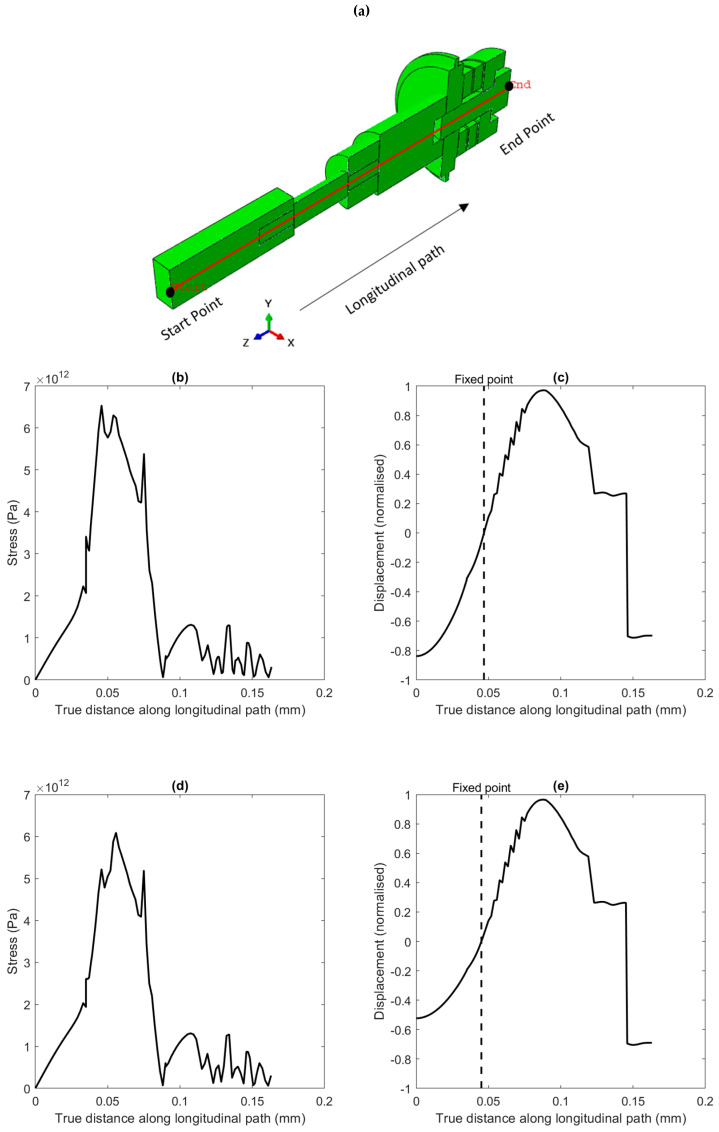
(**a**) A longitudinal path through the workpiece and the ultrasonic vibration-assisted milling device, (**b**) von Mises stress distribution along the path (Al 7075 workpiece), (**c**) normalised displacement distribution along the path (Al 7075 workpiece), (**d**) von Mises stress distribution along the path (Ti-6Al-4V workpiece) and (**e**) normalised displacement distribution along the path (Ti-6Al-4V workpiece).

A secure connection was essential for effectively transmitting stress waves from the transducer to the workpiece through the ultrasonic vibration-assisted milling device. Figure 5a depicts the longitudinal path, including the workpiece and device, as analysed through FEA methods to evaluate the mechanical behaviour at the horn-workpiece fixation point. Stress waves are continuously transmitted from the horn to the workpiece, facilitating the identification of the optimal fixation point with maximum stress and minimal displacement, as illustrated in Figure 5a,b. The propagation speed c and wavelength λ of the stress waves, which are influenced by material properties [35], guide this identification.

### 2.3. FEA Model Validation

**Figure 6:**
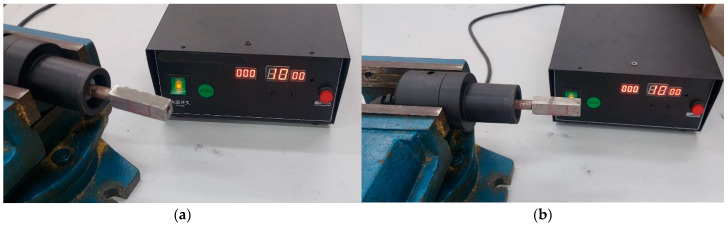
(**a**) Iron powder distributed along the workpiece in the initial position and (**b**) iron powder concentrated at the nodal plane after applying vibration.

The experimental measurements of the vibration amplitude and the validation of the FEA results by checking resonance frequency and nodal plan position were performed according to a technique proposed by Baraya et al. [35]. The transducer components, including the stepped horn, were manufactured and assembled for experimental validation. The workpiece was attached to the transducer through the designed connector. Then, the transducer was connected to the ultrasonic generator, and iron powder was distributed along the workpiece, as shown in Figure 6a.

The nodal plane location was validated using the iron powder, as depicted in Figure 5c,e. When the ultrasonic generator started to send the vibration waves to the transducer, the iron powder moved along the workpiece, responding to the vibration waves except at the nodal position, as shown in Figure 6b. This experimental observation confirmed that the nodal plane location corresponded closely with the predictions from the FEA.

## 3. Experimental Setup

In the current study, two workpiece materials were used: Al 7075, measuring 15 mm × 50 mm × 12 mm and Ti-6Al-4V titanium alloy, measuring 15 mm × 50 mm × 10 mm. These materials were subjected to CM and USVAM. Two different cutting tools were used: (S822) a 4 mm diameter carbide endmill with two flutes was used for Al 7075 workpieces, while (S260) a 4 mm diameter carbide endmill with four flutes was used for Ti-6Al-4V workpieces (Dormer Pramet, Sumperk, Olomoucky kraj, Czech Republic). The chemical and mechanical properties of the workpieces are listed in Table 3 and Table 4, respectively.

The experimental work was conducted using an Extron Vertical Milling Centre machine (CENTROID, Howard, PA 16841 USA). The vibration generation system was comprised of an ultrasonic generator unit with maximum power rating of 1 kW operating at a frequency of 28 kHz, a piezoelectric transducer and a booster to amplify the vibration amplitude. Vibration waves were transmitted to the workpiece through this setup during the cutting process.

**Figure 7:**
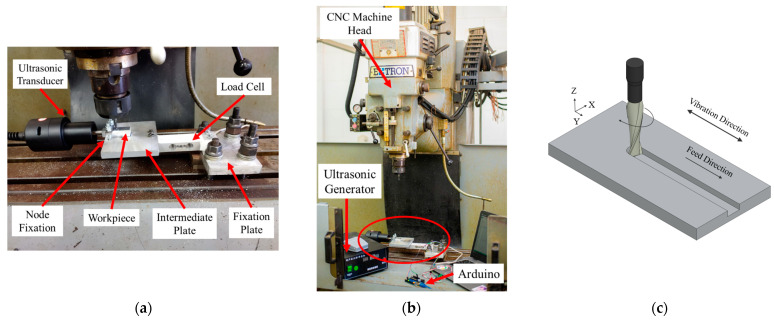
(**a**) Vibration system equipment, (**b**) complete setup with force-measurement system and ultrasonic components and (**c**) vibration direction during the milling process.

During the machining experiments, the axial cutting force was measured using a Wagezellen load cell model 102, measuring forces up to a capacity of 500 N. The mechanical loads were converted into an electrical charge by a piezoelectric sensor. This electrical charge was processed and recorded as force units by a programmed Arduino Uno (Somerville, Somerville, MA, US), as shown in Figure 7b. Arduino Uno interfaced with the sensor and converted analogue signals into digital formats for accurate force measurement and data recording. The surface roughness of the machined slots was assessed in order to compare the slotting process performance in CM and USM. During this process, the average roughness (Ra) and average maximum height of the profile (Rz) values were measured using a Surface Roughness Tester TR-200 (Bowers Group, Albany Court, Albany Park, Camberley, UK).

In this context, the Ra surface roughness unit was micrometres (µm), where Ra stands for “arithmetic average roughness” and measures the average deviation of the surface profile from the mean line over a specified length. Rz in micrometres represented the average maximum height of the profile and was calculated by taking the average of the vertical distance between the highest peak and the lowest valley in each sampling length and then averaging these values.

For each slot, three measurements were taken along a 4 mm segment of the slot length to ensure accuracy and consistency in the roughness evaluation. This method provided a detailed comparison of the surface finish quality achieved by each milling technique.

### 3.1. Experimental Design Methodologies

The experimental study aimed to optimise the milling process by investigating various input parameters. The input parameters examined were the depth of cut (mm), feed motion (mm/min) and spindle speed (rpm). Preliminary experiments were conducted to establish suitable upper and lower levels for these parameters. Based on the results of these preliminary tests and data from the literature, the parameter levels for the comprehensive set of experiments were determined. The total number of tests conducted was 18 experiments with 3 replications for each workpiece. The selected milling parameters and their levels are listed in Table 5. All experiments were performed under dry-cutting conditions.

### 3.2. SVR Model Construction

Support Vector Regression (SVR) is a Support Vector Machine (SVM) tailored to predict continuous values rather than classify data. While using SVR, the goal was to find a function that predicts surface roughness and cutting force from depth of cut, cutting and feed speeds with controlled assisted vibration within the smallest margin of error while keeping the model simple. As the most effective way to compare the performance of different AI-based algorithms is to cross-validate them, SVR was initially tested in the current study following a random 70%, 15% and 15% data partition strategy for training, validation and testing. Fifty-four data points were used in building the SVR model for each material (Al and Ti). It is crucial in this context to refer to the randomisation selection used for the training, validation and testing data as “pseudo-random selection” or “deterministic selection”, as the seed was fixed to a single value of 42 that controlled the random value generator. This value was selected after running that model with multiple seeds and identifying the seed that gave the best average accuracy across all AI models. A fixed seed approach was used across the study to ensure reproducibility and comparability among the results, which means that while the selection mimics randomness, the results can be exactly replicated in subsequent runs.

The SVR model was built and trained to predict the output variables, employing a Gaussian kernel using the MATLAB Deep Learning Toolbox “fitrsvm” function. The Gaussian function was selected as a kernel for its capability to capture nonlinear relationships between inputs and outputs. The kernel scale was set to “auto”, which automatically adjusted the kernel function’s influence by its width (3.0932 in this case, with 26 iterations), and the input values were standardised before training, ensuring that each value contributes equally to the prediction process. After being trained, the SVR model predicted the two outputs. The accuracy of these predictions was evaluated using two key metrics: Root Mean Square Error (RMSE) and Mean Squared Error (MSE). The RMSE provided an average measure of the prediction error magnitude. At the same time, the MSE offered insight into the model precision by averaging the squared differences between the predicted and true values.

### 3.3. ANN Model Construction

This section describes the implementation of an ANN in MATLAB Deep Learning Toolbox for predicting target variables (surface roughness and cutting force) from input features (depth of cut, cutting and feed speeds in addition to on/off assisted vibration). Once more, a subtotal of 54 data points was used for each material, with 108 total data points.

The process started with data preparation, where the input features and target variables were rearranged to match the required dimensions for the ANN and stored as ANN inputs and outputs. Then, a feedforward ANN with 10 neurons in its 2 hidden layers was created using the “fitnet” function. This setup was chosen to balance network complexity and performance. The training parameters procedure was achieved by setting the network configuration for 100 epochs with a learning rate of 0.01, optimising the training process using the Levenberg–Marquardt algorithm handled by the “train” function. The activation function used was the sigmoid function “tansig” in the hidden layers and a linear function “purelin” in the output layer.

**Figure 8:**
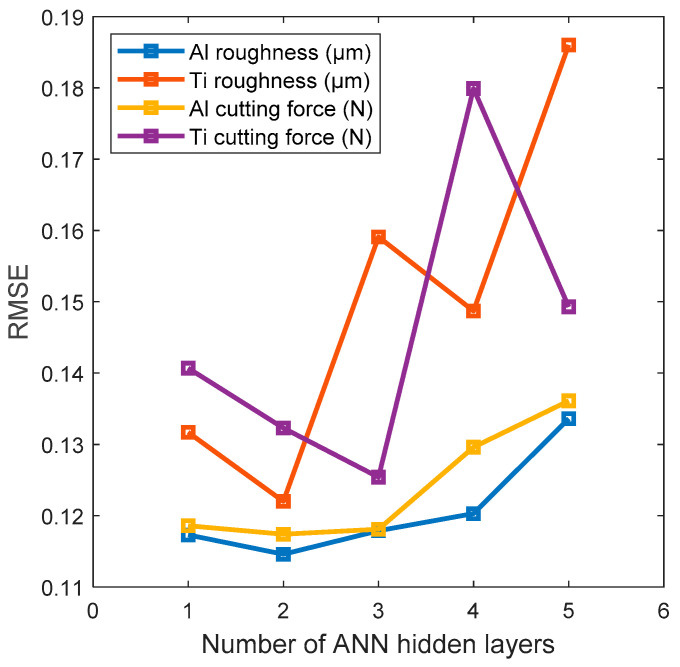
The process for selecting the number of hidden ANN layers was based on the overall RMSE error minimisation, where two layers were the best.

**Figure 9:**
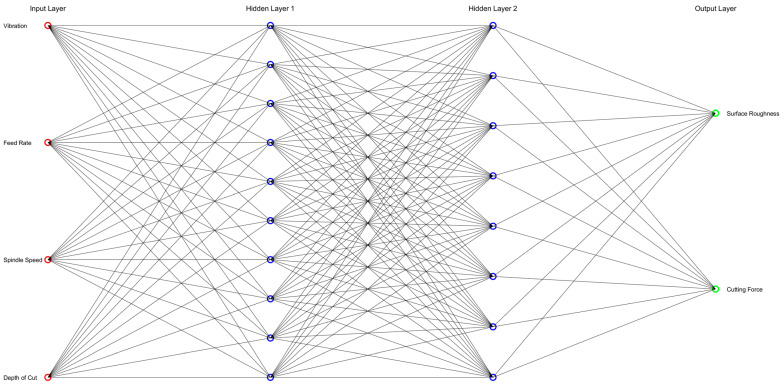
A simplified representation of the architecture of the ANN was built within the current study. Input neurons are coloured red, output neurons are green, and hidden layer neurons are blue. For simplicity, this figure does not display weights, bias or the activation function.

The number of hidden layers was set by starting with a single hidden layer and progressively increasing the number of hidden layers to five while evaluating the performance through the overall sum of Root Mean Squared error (RMSE) values. The outcome of this process indicated that two hidden layers were the best choice, as the lowest overall RMSE was recorded with them. The overfitting error was observed when more layers were tested (Figure 8); hence, the ANN model was structured as in Figure 9.

The dataset was randomly divided into 70% for training, 15% for validation and 15% for testing, ensuring a good mix and preventing overfitting. The network was trained using the prepared inputs and outputs, adjusting weights to minimise prediction errors. After training, the network was used to predict outputs based on the input data. In this case, prediction errors for the first and second output components, which were surface roughness and cutting force, were calculated. Finally, the RMSE was computed for each to evaluate network performance. RMSE was used to assess performance as it measures the average prediction error magnitude, reflecting variance and bias. RMSE is sensitive to large errors, making it an effective metric for identifying weighty prediction deviations. Additionally, its straightforward interpretation as the average deviation from actual values facilitates comparison across different models.

## 4. Results and Discussion

This section presents the outcomes of the slotting experiments conducted on the 7075 aluminium alloy and Ti-6Al-4V titanium alloy using CM and USVAM techniques. The focus was on the axial cutting force and the surface roughness of the slot button area.

### 4.1. Axial Cutting Force

**Figure 10:**
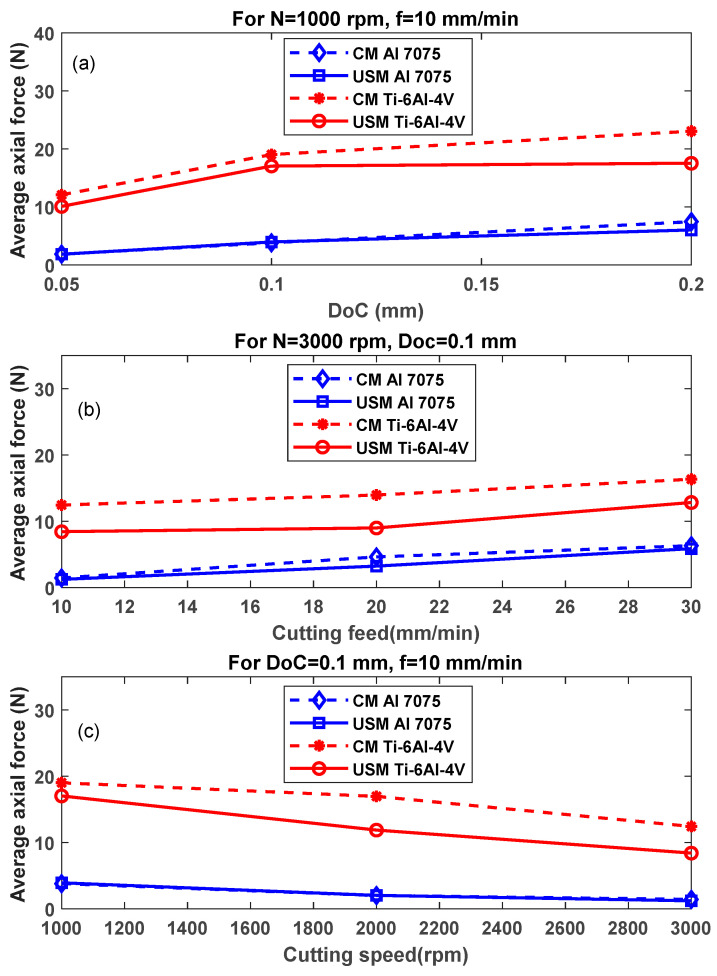
Average axial milling force verses (**a**) DoC (N = 1000 rpm, f = 10 mm/min), (**b**) cutting feed (N = 3000 rpm, DoC = 0.1 mm) and (**c**) cutting speed (DoC = 0.1 mm, f = 10 mm/min).

The axial cutting force data collected from the experiments reveal intriguing insights into the influence of USVAM on machining performance. Figure 10 summarises the average cutting forces recorded for the two investigated materials and milling techniques.

The results indicate a noticeable reduction in the axial force when US vibration is introduced for the Ti-6Al-4V alloy. Remarkably, no significant reduction was found for the Al 7075 alloy under any conditions. This phenomenon is likely attributed to the highly plastic nature of Al 7075 alloy, causing a large amount of vibration to be damped by the workpiece itself [43]. Hence, the interrupted cutting mechanism is not employed as expected. Increasing depth of cut (DoC) and cutting feed rates led to a rising trend in the axial force (see Figure 10a,b) as higher DoC and cutting feed causes an increase in the contact area between tool/workpiece interface, causing higher exerted force even when applying USM. Additionally, for the Ti-6Al-4V alloy, it could be seen that the axial force was reduced significantly by around 25% (at N = 1000 rpm, f = 10 mm/min and DoC = 0.2 mm) and by slightly more than one-third (at N = 3000 rpm, f = 20 mm/min and DoC = 0.1 mm). Figure 10c depicts a declining behaviour as the cutting speed increases; this occurs as higher cutting speed causes higher linear cutting velocity and hence higher shear rate as well as heat generation, which causes a thermal softening for the material [44] and decreases its shear strength, leading to a lower exerted force that USM can even reduce by nearly one third (at N = 2000 rpm, f = 10 mm/min and DoC = 0.1 mm) for the Ti-6Al-4V alloy.

### 4.2. Surface Roughness

**Figure 11:**
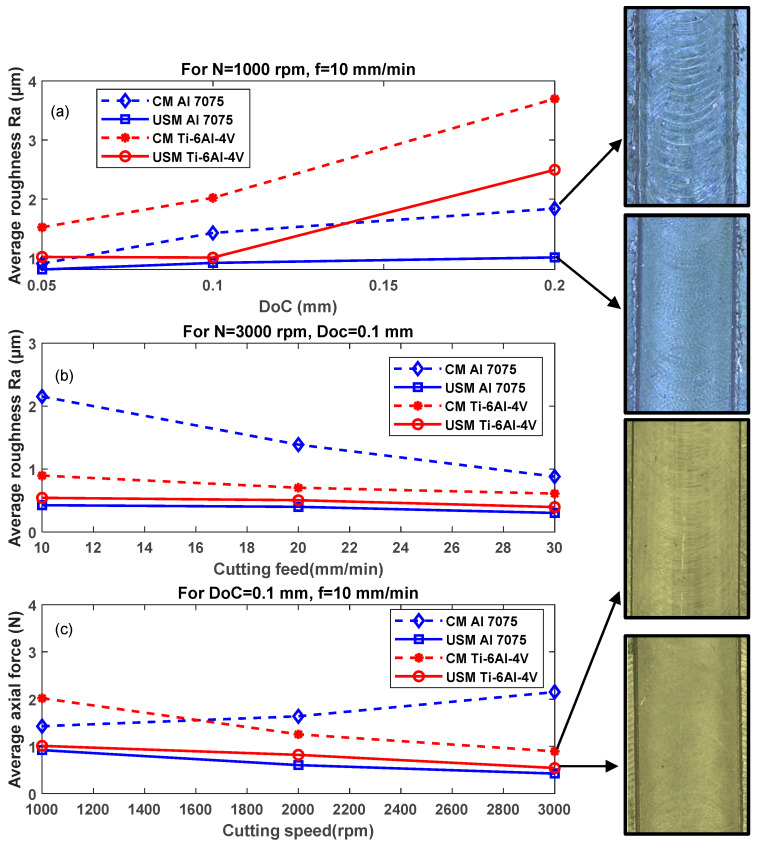
Average surface roughness verses (**a**) DoC (N = 1000 rpm, f = 10 mm/min), (**b**) cutting feed (N = 3000 rpm, DoC = 0.1 mm) and (**c**) cutting speed (DoC = 0.1 mm, f = 10 mm/min).

Surface roughness is a critical factor in determining product quality. The average surface roughness values measured for the slot bottom area of both materials and both milling techniques are summarised in Figure 11. Generally, applying US vibration led to an improvement in the surface roughness for both investigated materials. Figure 11a shows a rising trend of surface roughness as DoC increases either with or without US vibration; additionally, it could be seen that the US effect is higher as DoC increases to reach around 50% and 80% surface roughness reduction (at N = 1000 rpm, f = 10 mm/min and DoC = 0.2 mm) for the titanium and aluminium alloys, respectively. On the other side, higher cutting feed reduces the machining time and the friction time between the tool and the workpiece, leading to a better surface quality. According to Figure 11b, introducing vibrations improves the surface roughness at a lower cutting feed rate. However, this impact is decreased by increasing the cutting feed. The reason for this is that the application of USM at high levels of cutting feed does not lead to the interrupted cutting mechanism rather than additional applied cutting forces due to an extra contact between the cutting tool and the workpiece resulting from the application of vibration [45] (see the rising trend in Figure 4b). Similarly, based on Figure 11c, the reduction in surface roughness was significant for both materials as it reached a peak value for Ti-6Al-4V by nearly 100% (at N = 1000 rpm, f = 10 mm/min and DoC = 0.1 mm) and for Al 7075 by approximately 4 times (at N = 3000 rpm, f = 10 mm/min and DoC = 0.1 mm).

### 4.3. Tool Wear

**Figure 12:**
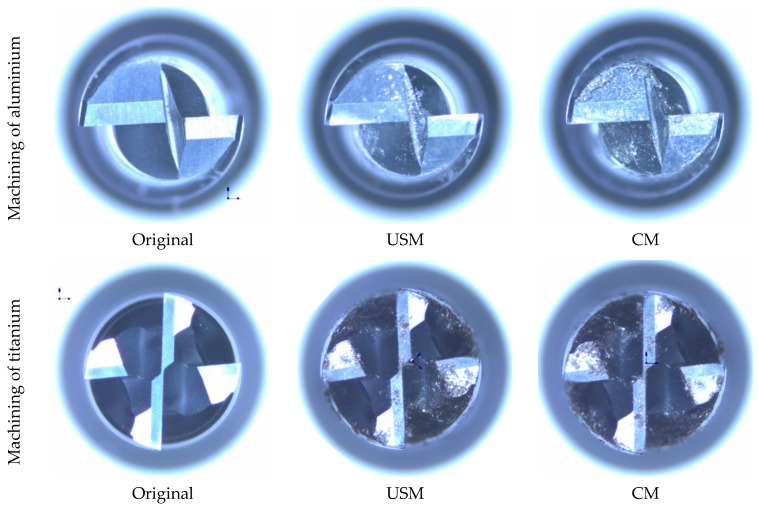
Optical microscope images for the cutting tool at its original state, after USM and after CM for aluminium and titanium alloys.

Tool wear criteria such as flank wear, tool edge radius and tool diameter are crucial in assessing tool life. These criteria depend on several factors and significantly influence cutting forces, surface quality and burr formation. This study used a two-flute end mill to machinate the Al 7075 alloy, while a four-flute end mill was employed for the Ti-6Al-4V alloy. As shown in Figure 12, tool wear is more pronounced in conventional milling (CM) than in USVAM for both aluminium and titanium workpieces.

**Figure 13:**
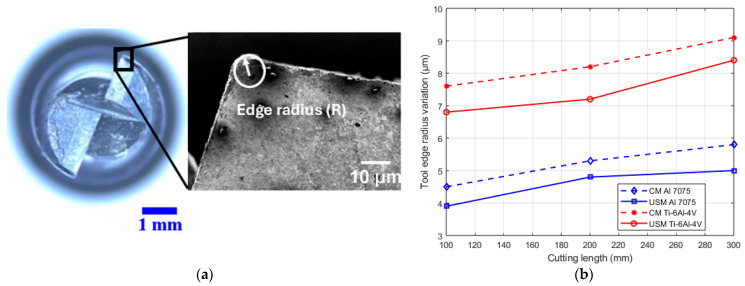
The measurement method of the tool edge radius (**a**,**b**) tool edge radius variation under different milling conditions and cutting lengths for aluminium and titanium alloys.

To evaluate tool wear, the geometry of the end mills was examined three times across cutting lengths up to 300 mm using a scanning electron microscope (SEM) (Figure 13a). The cutting edge radius was measured as a wear criterion for both materials, with each value measured three times to minimise error. The tools were also cleaned through a puff of compressed air before inspection after each cutting condition. The results, shown in Figure 13b, indicate that the cutting-edge radius increases in both CM and USM. However, when vibration was applied, the wear rate of the cutting-edge radius slowed down. For aluminium under CM, the edge radius increased from 4.5 to 5.8 µm as the cutting length extended from 100 to 300 mm. With vibration applied, this value decreased to 5 µm at the maximum cutting length, representing a reduction of approximately 16%. A similar trend was observed for titanium, with a lower reduction rate of 8% after vibration was applied. This suggests that vibration-assisted milling suppresses the blunting of the cutting edge, leading to longer tool life. The benefits of ultrasonic-assisted machining can be attributed to the enhanced material shear slip and the effective inhibition of chip sticking during chip separation, particularly in titanium [46]. This process not only facilitates more efficient chip discharge but also requires less cutting force, resulting in lower temperatures.

### 4.4. AI-Based Models Performance

Obtaining the correct cutting parameters in metalworking is essential for making the process efficient and ensuring high-quality results. Traditionally, finding comprehensive ranges of these parameters involves a lot of trial and error and extensive experimental testing, which can be both time-consuming and expensive. However, with the advent of ML, there was an innovative way to tackle this challenge. This is accomplished by running a limited number of well-designed experiments and predicting the broad feasible range of parameters through AI-based algorithms like SVR or ANN.

Table 6 presents a comparative analysis of the results of using two AI-based ML algorithms, ANN and SVR, in predicting surface roughness and cutting force for Al and Ti. The evaluation metrics used to assess model performance are RMSE and MSE.

**Figure 14:**
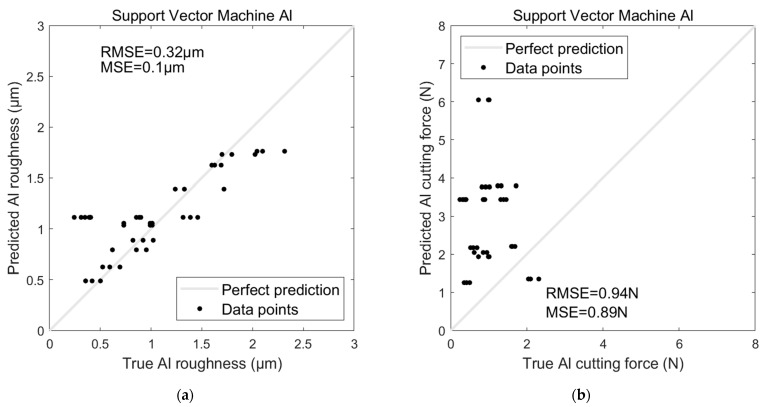
SVR model for predicting Al (**a**) surface roughness and (**b**) cutting force.

**Figure 15:**
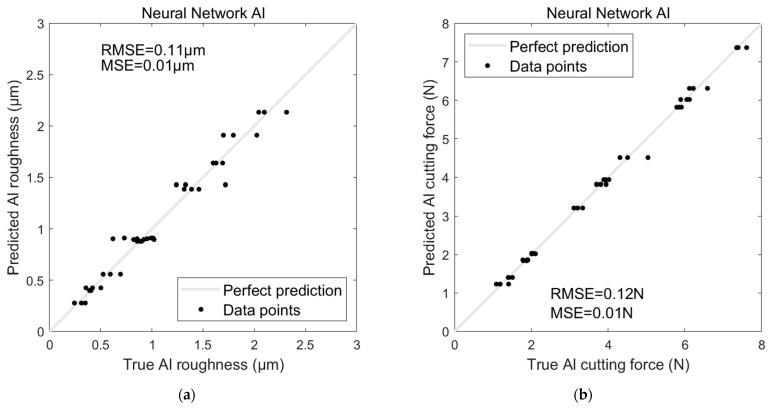
ANN model for predicting Al (**a**) surface roughness and (**b**) cutting force.

Regarding the AI-based models’ performances on Al, the ANN model significantly outperformed the SVR model when predicting surface roughness for Al (see Figure 14a and Figure 15a). The ANN achieved an RMSE of 0.11 µm and an MSE of 0.01 µm, indicating a high level of accuracy and minimal error in its predictions. In contrast, the SVR model exhibited a notably higher RMSE of 0.32 µm and an MSE of 0.1 µm, suggesting that it struggled to capture the underlying patterns in the data as effectively as the ANN.

Similarly, the ANN model again demonstrated superior performance for predicting cutting force (see Figure 14b and Figure 15b), with an RMSE of 0.12 N and an MSE of 0.01 N. The SVR model, however, showed a significant drop in accuracy, with an RMSE of 0.94 N and an MSE of 0.89 N. These results highlight the ANN’s ability to make more precise predictions than the SVR model, particularly for the Al dataset.

**Figure 16:**
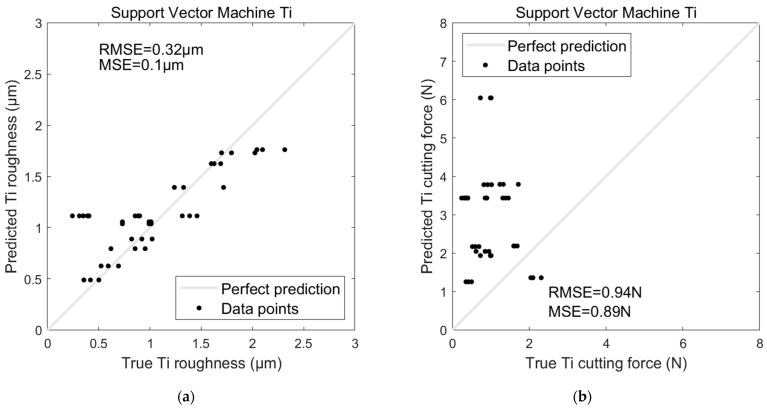
SVR model for predicting Ti (**a**) surface roughness and (**b**) cutting force.

**Figure 17:**
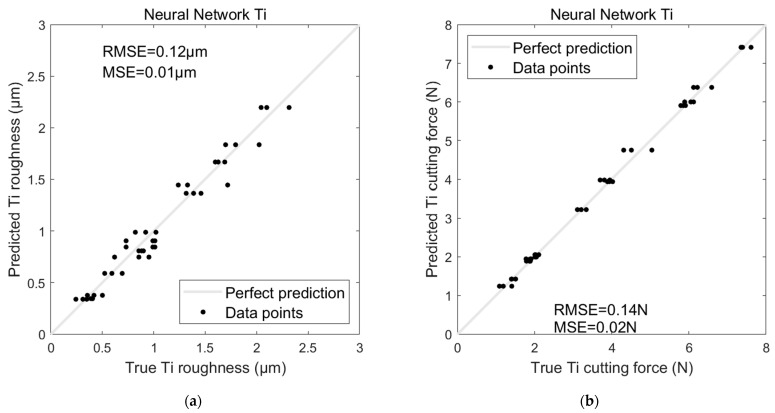
ANN model for predicting Ti (**a**) surface roughness and (**b**) cutting force.

The performance trends observed with Al were also evident in the Ti dataset regarding the AI-based models’ performances on Ti. The ANN model maintained its advantage, producing an RMSE of 0.12 µm and an MSE of 0.01 µm for roughness prediction, compared to the SVR model’s RMSE of 0.32 µm and MSE of 0.1 µm (see Figure 16a and Figure 17a). For cutting force prediction, the ANN achieved an RMSE of 0.14 N and an MSE of 0.02 N, while the SVR again performed less favourably with an RMSE of 0.94 N and an MSE of 0.89 N (see Figure 16b and Figure 17b).

Validated by the experimental findings, the ANN consistently outperformed the SVR model across materials and prediction tasks. The lower RMSE and MSE values associated with the ANN indicated that it was better suited to capturing the complex relationships within the data, resulting in more accurate and reliable predictions. In contrast, while capable of making predictions, the SVR model did so with considerably more error, particularly in predicting cutting force. This comparison underscored the effectiveness of ANN in tasks requiring high precision. It suggested that ANN was a more robust choice for predicting material properties like roughness and cutting force in manufacturing processes.

## 5. Conclusions

The results of this study underscore the potential of ultrasonic vibration-assisted milling as a practical approach for enhancing machining efficiency and product quality. The reduction in cutting forces and improvements in surface finish indicate the technique’s viability in both brittle and ductile material scenarios. This research contributes to the broader understanding of advanced machining technologies and offers a foundation for further exploration and implementation.

The introduction of ultrasonic waves causes a noticeable decrease in the axial cutting force by up to 30% in the case of Ti-6Al-4V alloy. However, the application of ultrasonic machining to the Al 7075 alloy did not significantly impact the axial cutting force, likely due to the high plasticity of the aluminium alloy, which causes vibration damping. Surface roughness was enhanced for both materials with the application of ultrasonic waves, with a maximum reduction of approximately 50% for Ti-6Al-4V and 40% for Al 7075. Additionally, increasing the feed rate resulted in a less rough surface in the case of USM, as applying ultrasonic waves at high cutting feed rates does not lead to an interrupted cutting mechanism but instead increases the applied cutting forces. Furthermore, cutting tools for both materials experienced higher wear in CM than USM.

Like most studies in vibration-assessed cutting, the current study also had experimental and numerical limitations. Experimentally, since the ultrasonic generator has a limited frequency range, this study used a single resonance amplitude of 8 µm, the maximum possible amplitude at the resonance. Numerically, a proper randomisation process in selecting AI training, validation and testing data was not practical as it makes the results irreproducible. Therefore, a MATLAB pseudo-random number generator was employed after running all models with multiple seeds and selecting the one that gave the best accuracy.

Considering the consumed time and the high cost of experimental testing for a wide range of metal cutting parameters, advanced methods, such as AI-based algorithms, are needed to accurately predict the effect of different cutting parameters and their companions with a limited experimental range of testing. Compared to SVR, the ANN has proven effective in predicting cutting patterns and relationships, making it perfect for applications in metal cutting assisted by vibration. This study explored how a well-structured ANN can better predict important cutting parameters such as the cutting force and surface roughness than SVR. The ANN outperformed SVR because it better captured the nonlinear relationships between the machining parameters and outcomes. The ANN’s flexible structure allowed it to model these interactions accurately, while SVR struggled. Additionally, ANN’s ability to generalise across different conditions made it a good choice for predicting outcomes in ultrasonic vibration-assisted milling.

In conclusion, by utilising experimental data and advanced methods, AI-based ANNs provide accurate forecasts without requiring extensive full-ranged resolution physical experimentations, saving time and costs while optimising the metal-cutting process for better efficiency.

## Figures and Tables

**Table 1:** Properties of the piezoelectric elements.

Item Code	BJUPR
Dimensions (mm)	35 × 15 × 5
Density (Kg/m^3^)	7.7
capacity (p Farad)	1720
Piezoelectricity (10^−12^ Coulomb/Newton)	D_33_ = 240, d_31_ = −95, d_15_ = 380
Dielectricity (ε_0_ = 8.85 × 10^−12^ Farad/meter)	ε_33_^S^ /ε_0_ = 540, ε_11_^S^/ε_0_ = 800
Elastic compliance (10^−12^ meter^2^/Newton)	S_11_^E^ = 11.4, S_33_^E^ = 13.7

**Table 2:** Physical properties of transducer component materials.

No.	Material	Young’s Modulus (GPa)	Density (Kg/m^3^)	Poisson’s Ratio	Wave Velocity (m/s)	Characteristic Acoustic Impedance (10^6^·Ns/m^3^)
1	Aluminium (5083)	70.3	2660	0.33	5140	13.67
2	Steel (AISI 1045)	200	7870	0.3	5040	39.7
3	Piezoelectric	73	7700	-	3080	23.72
4	Copper	115	8900	0.31	3595	31.9

**Table 3:** Chemical composition (wt.%) of Al-7075 and Ti-6Al-4V [38,39].

**Al-7075**									
**Element**	**Zn**	**Mg**	**Cu**	**Si**	**Mn**	**Cr**	**Fe**	**Ti**	**Al**
wt.%	3.25	1.9	1.8	0.5	0.4	0. 2	0.5	0.15	Balanced
**Ti-6Al-4V**									
**Element**	**Al**	**V**	**Fe**	**C**	**N**	**H**	**O**	**Ti**	
wt.%	5.5~6.8	3.5~4.5	≤0.30	≤0.08	≤0.05	≤0.015	≤0.20	Balanced	

**Table 4:** Mechanical properties of Al-7075 and Ti-6Al-4V [39,40,41,42].

Property	Elongation (%)	Hardness (HV)	Tensile Strength (MPa)	Elastic Modulus (GPa)	Yield Strength (MPa)	Poisson Ratio
Al-7075	11	175	570	72	505	0.33
Ti-6Al-4V	14.5	350	1150	125	950	0.342

**Table 5:** Experimental parameters for Al 7075 and Ti-6Al-4V titanium alloy workpieces.

Levels	Machining Condition			
	Depth of Cut (mm)	Feed (mm/min)	Spindle Speed (rpm)	Ultrasonic Vibration
1	0.05	10	1000	ON/OFF
2	0.1	20	2000	ON/OFF
3	0.2	30	3000	ON/OFF

**Table 6:** Comparison of AI-based model performances.

		Roughness	Roughness	Cutting Force	Cutting Force
Material	AI-Based Model	RMSE (µm)	MSE (µm)	RMSE (N)	MSE (N)
Al	SVR	0.32	0.1	0.94	0.89
Al	ANN	0.11	0.01	0.12	0.01
Ti	SVR	0.32	0.1	0.94	0.89
Ti	ANN	0.12	0.01	0.14	0.02

## Data Availability

The data presented in this study are available on request from the corresponding author. The data are not publicly available due to considerations regarding possible future commercialisation.
